# Evaluation of infection control effectiveness in healthcare workers' practices using the ASK model

**DOI:** 10.3389/fpubh.2026.1721061

**Published:** 2026-03-02

**Authors:** Lili Jia, Shuo Bai, Fang Zhang, Rong Chen, Huimin Huang

**Affiliations:** 1 Department of Pediatrics, Jinling Hospital, Affiliated Hospital of Medical School, Nanjing University, Nanjing, China; 2 Department of Cardiology, Jinling Hospital, Affiliated Hospital of Medical School, Nanjing University, Nanjing, China; 3 Department of Disease Prevention and Control, Jinling Hospital, Affiliated Hospital of Medical School, Nanjing University, Nanjing, China

**Keywords:** ASK model, cleaning staff, evaluation, infection control, training

## Abstract

**Objective:**

This study evaluated the impact of ASK model-based training on infection control practices among hospital cleaning staff.

**Methods:**

We recruited 31 cleaning staff from the internal medicine department. A tailored ASK training system was implemented, comparing pre- and post-training performance in key infection control procedures, theoretical knowledge, and ATP-based cleaning efficacy.

**Results:**

Post-training hand hygiene compliance increased from 30.30%to 83.62% (*p* < 0.05). Knowledge assessment pass rates improved from 45.20 to 83.90% (hand hygiene) and 38.70 to 71.00% (cleaning-related knowledge). ATP pass rates for hand hygiene and environmental surfaces also improved significantly (both *p* < 0.05).

**Conclusion:**

ASK model training effectively enhances cleaning staff's knowledge, skills, attitude, and infection control performance, suggesting a useful framework for future training programs.

## Background

Preventing and controlling hospital-acquired infections (HAIs) is essential for ensuring patient safety and enhancing the overall quality of healthcare (1). The sanitation of hospital environments is closely linked to the incidence of HAIs (2), with timely and effective cleaning serving as a foundational measure for infection prevention and control (3). Due to their specific roles and work settings, hospital cleaning staff are not only at risk of occupational exposure but may also act as potential sources or vectors of transmission (4). Therefore, they require certain professional competencies, including relevant knowledge and skills, to perform their duties safely and effectively (5). Currently, training for cleaning personnel is commonly provided by outsourced contractors or hospital logistics departments (6); however, such training often lacks a systematic approach to developing attitudes, skills, and knowledge, as well as sustained supervision mechanisms (7). The ASK (Attitude, Skill, Knowledge) model, also referred to as the “Success Competency Model”, aims to improve professional competence through the integrated development of these three core dimensions. Guided by this framework, we implemented a structured training program for cleaning staff and assessed its impact through behavior-based evaluations, demonstrating promising outcomes (8). Specifically, cleaning staff are at risk of occupational exposures such as needlestick injuries, contact with blood or body fluids, and exposure to multidrug-resistant organisms (MDROs), all of which underscore the necessity for systematic training in infection control principles and practices. While knowledge is often emphasized in traditional training programs, it is the cultivation of a proactive attitude and the development of practical skills that are crucial for driving consistent behavioral compliance in daily practice. Guided by this framework, we implemented a structured training program for cleaning staff and assessed its impact through behavior-based evaluations, including direct observation of hand hygiene and cleaning compliance, theoretical knowledge tests, and objective ATP monitoring of cleaning efficacy. The promising outcomes refer to the observed improvements in these indicators, such as increased compliance rates, higher test scores, and improved ATP pass rates, as detailed in the **Results** section.

## Methods

### Research subjects

This study included cleaning staff working in the internal medicine department of our hospital between September and December 2022. Inclusion criteria required a minimum of 3 months of employment at the hospital. Cleaners who were on leave or declined to participate were excluded. A total of 31 cleaners met the criteria and were enrolled in the study, comprising 11 males and 20 females, all aged 55 years or above. Educational background was generally low: 18 (58.06%) had completed primary school or less, 12 (38.71%) had junior high school education, and one (3.23%) had senior high school education. Most participants were previously laid-off workers, homemakers, or unemployed, and none had worked for a professional cleaning company prior to joining the hospital. In terms of work experience, 26 (83.87%) had been employed at the hospital for more than 1 year. Regarding prior training exposure, five had never received any hospital infection control training, eight had attended once, and 18 had participated in training two or more times (Table 1).

**Table 1:** Basic information of cleaning staff.

**Variables**	**Clusters**	**Number**	**Percentage (%)**
Sex	Male	11	35.48%
	Female	20	64.52%
Education level	Primary school and below	18	58.06%
	Junior high school	12	38.71%
	Senior high school	1	3.23%
Work Experience	Professional cleaning company	0	0%
Years of working	Less than one year	5	16.13%
	One year or more	26	83.87%
Number of training participations	None	5	16.13%
	Once	8	25.81%
	2 times or more	18	58.06%

The sample size was determined by the total number of eligible cleaning staff in the internal medicine department during the study period. Although relatively small, this sample represents the entire target population within the designated clinical setting. We acknowledge that the limited sample size may affect statistical power and the generalizability of findings to other contexts.

### Establish an infection control training team based on the ASK model

A three-tier training team was established, structured as “dedicated infection control personnel—core members of the infection control subcommittee—clinical infection control supervisors”. This team consisted of 10 members in total: three from the Disease Prevention and Control Department, two head nurses from the infection control subcommittee, one nurse from the infection control subcommittee, and four clinical infection control supervisors. Personnel from the Disease Prevention and Control Department were primarily responsible for formulating teaching objectives, providing in-depth guidance, and monitoring training effectiveness. The head nurse of the infection control team served as the lead instructor, overseeing training assessments and organizing team-building activities. The infection control team nurse acted as the teaching coordinator, managing curriculum planning, tracking progress, facilitating communication, and collecting data. Clinical infection control supervisors conducted demonstration rounds, performed on-site supervision, and carried out evaluations. All team members held professional titles at the supervisor level or above, possessed over 10 years of relevant experience, and had completed standardized ASK model training prior to the study implementation. The training program was conducted over a period of 4 weeks, with structured sessions held twice weekly (each session lasting 60–90 min). The training team consisted of 10 members as described previously. To ensure adherence to the ASK model, all trainers participated in a standardized train-the-trainer workshop prior to intervention delivery. Training fidelity was monitored through weekly team meetings, review of session checklists, and direct observation of randomly selected sessions by the lead infection control nurse. Attendance was mandatory for all cleaning staff, and all participants completed the full training schedule.

### Establishing an infection control training system based on the ASK model

Based on the ASK model and informed by the specific characteristics of the cleaning staff and the current landscape of infection control training, the research team established a three-tiered supervision and training framework. This system involved dedicated infection control personnel, infection control nursing supervisors, and clinical infection control monitors working in coordination (9).

#### Knowledge (K) component

Teaching objectives were formulated using Bloom's Taxonomy, focusing on integrating cognitive, affective, and behavioral competencies tailored to the physiological, psychological, and social profiles of the cleaning staff. Four practical, infection control-specific skills essential to their role were selected. Hands-on, experiential tutorials for these skills were developed to form the knowledge core of the ASK-based curriculum. Training was delivered through diverse methods, including lectures, case discussions, and real-time instruction. Upon completion, all cleaners underwent a standardized assessment. Those who did not pass were required to repeat the training until competency was achieved. This component emphasized not only conceptual mastery but also the flexible application of knowledge in practice.

#### Attitude (A) component

This component aimed to cultivate a professional, patient-centered mindset, underscoring that environmental cleaning and disinfection are fundamental to ensuring safe and comfortable healthcare environments. The attitude framework was operationalized by organizing cleaners into four teams of 7–8 members, each led by a designated team leader who assisted infection control supervisors. Each Monday, the infection control nurse manager scheduled the week's hands-on training sessions. Using real clinical cases in a case-based teaching format, the critical importance of infection control was communicated, helping cleaners recognize the risks associated with inadequate cleaning. Clinical infection control supervisors then reinforced these concepts through on-site demonstrations and scenario-based guidance, facilitating the internalization of professional attitudes and their translation into consistent practice.

#### Skill (S) component

To build practical competency within the ASK framework, a weekly session was held every Friday for cleaning staff to share insights, technical experiences, and operational reflections from the week. Through structured scenario simulations and role-playing exercises, staff integrated infection control knowledge and attitudes into their daily routines. Clinical infection control supervisors provided close monitoring and feedback during these sessions, further deepening awareness and ensuring that infection control principles were authentically embedded into everyday cleaning practices.

### Evaluation indicators

The effectiveness of the training program was evaluated across three domains: 1) the compliance rate with key infection control procedures before and after training, 2) the pass rate on theoretical assessments of foundational infection control knowledge, and 3) the compliance rate of hand hygiene and environmental surface disinfection. These evaluations were implemented using a combination of theoretical testing, direct on-site observation of operational practices, and objective monitoring of cleaning and disinfection quality via ATP bioluminescence.

### Statistical methods

Data were analyzed using SPSS 22.0 software. Count data were expressed as frequencies and percentages. Intergroup comparisons were performed using the chi-square test or Fisher's exact test as appropriate. In addition to *p*-values, 95% confidence intervals (CIs) for proportions are reported where applicable. A *p*-value < 0.05 was considered statistically significant. Given the exploratory nature of this study and the interrelated outcome measures, we present unadjusted *p*-values but acknowledge the potential for inflated Type I error due to multiple comparisons.

## Results

The compliance rates for key infection control procedures before and after ASK model training are presented in Table 2. Prior to training, hand hygiene compliance among cleaning staff was 30.30%. Following training, this rate increased significantly to 83.62% (*p* < 0.05). The compliance rate for standardized cleaning and disinfection procedures also improved, rising from 74.07 to 90.65%; however, this change was not statistically significant (*p* = 0.505).

**Table 2:** Comparison of compliance rates for key infection control procedures among cleaning staff (*n*/%).

**Variables**	**Number of executions**	**Actual execution count**	**percent of pass**	**Monitoring frequency**	**Correct count**	**Percent of pass**
Before the training	198	60	30.30%	81	60	74.07%
After the training	519	434	83.62%	107	97	90.65%
X2			121.21			1.908
*P*			< 0.05			0.167

### Comparison of theoretical knowledge assessment pass rates before and after ASK model training

As shown in Table 3, the pass rate for hand hygiene knowledge among cleaning staff increased from 45.20% before training to 83.90% after training. Similarly, the pass rate for cleaning-related infection control knowledge rose from 38.7 to 71.0%. Both improvements were statistically significant (*p* < 0.05).

**Table 3:** Comparison of pass rates for theoretical assessment of infection control knowledge among cleaning staff (*n*/%).

**Variables**	**Number**	**The pass rate of the hand hygiene knowledge assessment**	**The pass rate of the cleaning and infection control knowledge assessment**
Before the training	31	14 (45.20)	12 (38.70)
After the training	31	26 (83.90)	22 (71.00)
X2		10.145	6.513
*P*		0.001	0.011

The pass rates for cleanliness and disinfection effectiveness before and after ASK model training are summarized in Table 4. Prior to the intervention, the ATP monitoring pass rate for hand disinfection among sanitation workers was 29.00%, which increased to 54.80% post-training. Similarly, the ATP monitoring pass rate for environmental surface cleaning and disinfection improved from 54.80% to 83.90%. Both increases were statistically significant (*p* < 0.05). ATP monitoring provides an objective measure of organic residue on surfaces and hands, with lower RLU values indicating better cleaning efficacy. In this study, the median RLU for hand hygiene decreased from 25.00 to 70.00, and for environmental surfaces from 60.00 to 35.50, reflecting improved cleaning and disinfection quality post-training. These reductions are clinically meaningful, as higher ATP levels are associated with increased risk of microbial contamination and potential transmission.

**Table 4:** Comparison of ATP monitoring pass rates for cleaning and disinfection effectiveness in sanitation work (*n*/%).

**Variables**	**Number**	**Hand hygiene ATP monitoring pass rate**	**RLU**	**The qualified rate of ATP monitoring for environmental surface cleaning and disinfection**	**RLU**
Before the training	31	9 (29.00)	25.00 (3.35–55.00)	17 (54.80)	60.00 (14.50–175.00)
After the training	31	17 (54.80)	70.00 (15.50–195.00)	26 (83.90)	35.50 (8.50–98.00)
X2		4.23	8.471	6.147	22.128
*P*		0.039	0.031	0.013	< 0.05

## Discussion

### Training based on the ASK model has enhanced the initiative and proactivity of cleaning staff

Cleaning staff play a vital role in hospital infection control, and their proactive engagement directly influences environmental hygiene (10). Many training approaches for this group have been explored. For instance, competency-based models and behavioral interventions often target specific skill gaps or practice changes (11), while multimodal training combines various methods to reinforce learning. However, traditional programs frequently integrate theoretical instruction for cleaning personnel with that for clinical staff, lacking content tailored to their specific roles and foundational knowledge levels. This generic approach can hinder comprehension and result in low initiative in daily practice.

The ASK model shares the holistic intent of competency frameworks but distinctively emphasizes the sequential and interdependent development of Attitude, Skill, and Knowledge. It acknowledges that while knowledge and operational skills can be acquired in the short term, fostering a professional, patient-centered attitude requires sustained and targeted cultivation. This focus on attitudinal development aligns with and extends principles from motivational and human-factor interventions, which seek to alter underlying perceptions and intentions to drive behavior (12). The development of such an internalized attitude in turn motivates staff to engage actively and adhere to procedures consistently. In this study, the marked increase in hand hygiene compliance—from 30.30% before training to 83.62% afterward—illustrates how integrating attitude-focused training within a structured model can effectively translate into sustained behavioral outcomes, a challenge often noted in training evaluations that rely solely on skill demonstration or knowledge testing.

### Training based on the ASK model to enhance infection control knowledge and operational skills

Hospital cleaning staff often have lower educational backgrounds, are generally older, and may experience challenges in acquiring new knowledge—a profile that necessitates tailored educational strategies. In China, a systematic and well-established training mechanism specifically for this group is currently lacking (13). Prior to employment, most cleaning personnel receive only basic procedural instruction from outsourcing companies, with limited emphasis on the underlying principles of infection control. These companies frequently prioritize cost efficiency and operational simplicity, often resulting in the inadequate implementation of fundamental infection control measures.

Existing training interventions for similar support staff populations have explored various approaches. Competency-based models typically outline specific skill and knowledge standards (14), while behavioral interventions often target discrete practice changes through feedback or incentives. Multimodal training, which combines methods like demonstration, practice, and reminder systems, has shown promise in improving adherence. Building upon these concepts, this study adopted the ASK model to construct a more holistic and structured training system that deliberately integrates and sequences three core dimensions: attitude, skills, and knowledge. Our system operationalized this framework by: formulating clear, behaviorally-anchored instructional objectives; selecting four representative infection control operations to illustrate key principles; facilitating interactive group brainstorming and experiential team activities; and deploying clinical infection control supervisors to provide continuous mentorship, demonstrations, and corrective feedback. Through scenario simulations, role-playing, guided reflection, and shared discussions, the training moved beyond rote learning to support the development of both practical proficiency and a humanistic, professional mindset (15).

This integrated, ASK-based approach facilitated a progressive “knowledge acquisition–attitude change–skill enhancement” cycle, enabling staff to more effectively internalize and apply their learning. Following the training, participants demonstrated markedly improved comprehension of core infection control concepts—including handwashing technique, hand hygiene indications, hand disinfection procedures, correct disinfectant dilution, and standardized surface cleaning methods. These outcomes underscore the model's effectiveness in translating structured training into measurable competencies, suggesting that its tripartite focus may address gaps left by narrower skill-based or knowledge-centric training programs reported in earlier studies (16).

### Training based on the ASK model to improve the quality of cleaning and disinfection work

Hospital environmental surfaces can harbor substantial numbers of pathogenic microorganisms, which may be transmitted via staff hands and contribute to cross-infection among patients. Effective hand hygiene and thorough environmental cleaning are therefore fundamental to preventing hospital-acquired infections, tasks largely carried out by cleaning staff (17). Prior to training, assessments indicated that staff often conflated medical hygiene standards with routine domestic cleaning, relying on visual assessment and personal habit rather than evidence-based protocols (18). This approach, coupled with limited awareness of the critical role their work plays in infection prevention, frequently resulted in inconsistent and suboptimal cleaning practices (19).

To address these competency gaps, the ASK model was implemented to reframe routine cleaning tasks into a structured, experiential learning process centered on knowledge, attitude, and skill development (20). This pathway-based approach transformed repetitive work into opportunities for guided practice, reflection, and internalization of principles, thereby motivating staff to proactively apply evidence-based methods. Post-training results demonstrated measurable improvements in both hand hygiene adherence and environmental disinfection efficacy, underscoring how integrating attitude and skill development with knowledge transfer can elevate the overall quality of cleaning practices.

#### Limitations of the study

This study adopted a before-after design to evaluate the impact of ASK model-based training on the infection control practices of cleaning staff. While the results showed significant improvements in multiple indicators, several limitations should be acknowledged.

Firstly, the absence of a control group limits the causal interpretation of the findings. Although we observed substantial improvements in hand hygiene compliance, theoretical knowledge, and ATP monitoring pass rates after training, we cannot rule out the influence of external factors such as seasonal variations in infection control awareness, concurrent hospital-wide hygiene campaigns, or the Hawthorne effect—where participants modify their behavior simply because they are under observation. Without a parallel control group that did not receive the training, it is challenging to attribute all observed changes solely to the ASK model intervention.

Secondly, the small sample size (*n* = 31) may affect statistical power and generalizability. The sample was constrained by the number of cleaning staff working in the internal medicine department during the study period. While the results are statistically significant for most indicators, the limited sample may reduce the robustness of subgroup analyses and limit extrapolation to other hospital settings or cleaner populations with different demographic profiles. Future multi-center studies with larger samples are warranted.

Thirdly, although we structured the training around the ASK framework, we acknowledge that increased supervision and observation during the study period could have independently influenced cleaner behavior. The presence of infection control monitors during assessments may have heightened staff attention to protocol adherence, potentially inflating post-training compliance rates.

Lastly, the use of multiple comparisons across several outcome indicators increases the risk of Type I error. While we reported *p*-values and confidence intervals, future studies might consider adjustment methods (e.g., Bonferroni correction) when evaluating multiple related outcomes.

Despite these limitations, the before–after design was chosen due to practical and ethical considerations within the hospital setting. Randomly assigning cleaning staff to a non-training group was deemed inappropriate given the critical role of infection control in patient safety. The study provides preliminary evidence that ASK-based training is feasible and associated with meaningful improvements in infection control metrics.

## Conclusion

This study developed and implemented a tailored ASK-model training system for hospital cleaning staff, grounded in Bloom's Taxonomy as the instructional framework. The training significantly enhanced cleaning personnel's infection control awareness, theoretical knowledge, and practical standardization. However, limitations in time, staffing, and resources confined the intervention to a single cohort within the internal medicine department. Future research should expand the sample size and extend to other clinical areas, further refine ASK-based training content, evaluate the long-term sustainability of outcomes, and explore its adaptability across varied healthcare settings. Such efforts will contribute to the development of innovative, competency-based training strategies for diverse hospital support staff.

## Data Availability

The original contributions presented in the study are included in the article/supplementary material, further inquiries can be directed to the corresponding author.
